# Masquerade Syndrome of Multicentre Primary Central Nervous System Lymphoma

**DOI:** 10.1155/2011/329857

**Published:** 2011-12-25

**Authors:** Silvana Guerriero, Ermete Giancipoli, Lorenza Ciracì, Giuseppe Ingravallo, Marcella Prete, Elisabetta Di Leo, Antonietta Cimmino, Nicola Cardascia

**Affiliations:** ^1^Department of Ophthalmology, University of Bari Medical School, 70124 Bari, Italy; ^2^Department of Pathology, University of Bari Medical School, 70124 Bari, Italy; ^3^Department of Internal Medicine and Clinical Oncology, University of Bari Medical School, 70124 Bari, Italy; ^4^Department of Ophthalmology and Otolaringology, University of Bari, 70124 Bari, Italy

## Abstract

*Purpose*. In Italy we say that the most unlucky things can happen to physicians when they get sick, despite the attention of colleagues. To confirm this rumor, we report the sad story of a surgeon with bilateral vitreitis and glaucoma unresponsive to traditional therapies. *Methods/Design*. Case report. *Results*. After one year of steroidal and immunosuppressive therapy, a vitrectomy, and a trabeculectomy for unresponsive bilateral vitreitis and glaucoma, MRI showed a multicentre primary central nervous system lymphoma, which was the underlying cause of the masquerade syndrome. *Conclusions*. All ophthalmologists and clinicians must be aware of masquerade syndromes, in order to avoid delays in diagnosis.

## 1. Introduction

In Italy we often say that doctors may be especially unlucky when they get sick, and that the strangest things can happen to them, despite the attention of colleagues. To confirm this rumor, we recount the sad story of an unfortunate colleague who suffered many tribulations and waited a year for a grim but not difficult diagnosis.

## 2. Case Report

In December 2009, a 44-year-old surgeon underwent bilateral phacoemulsification and IOL implantation for surgical correction of a 10-diopter myopia in a private clinic in northern Italy. Her natural visual acuity after surgery was 20/20 in both eyes.

**Figure 1:**
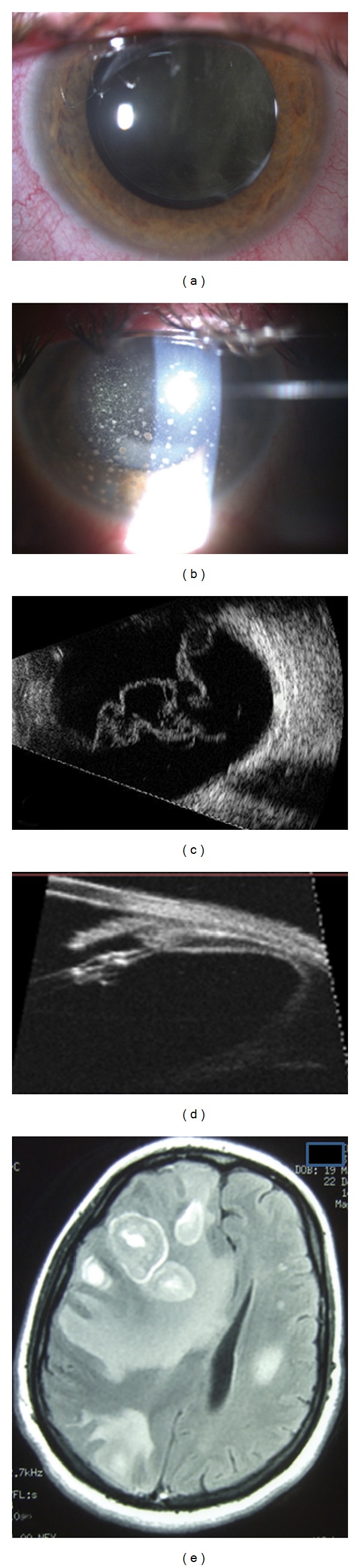
(a) Right eye showing a hazy vitreous humor. (b) Left eye showing an intense inflammation of the anterior chamber, with corneal precipitates. (c) Echographic examination of the vitreous in the right eye. (d) UBM examination of the ciliary body showing vitreous membranes adherent to the pars plana. (e) MR image showing a hypointense multifocal lesion occupying much of the right frontal and temporal lobe, the right occipital lobe, and the left parietal lobe.

Three months after surgery, she referred blurred vision in both eyes. A diagnosis of bilateral vitreous hemorrhage was made and she was referred for ultrasonography, which showed severe vitreitis, with intense flare cells and dense membranes (Figures 1(c) and 1(d)). At this time, her visual acuity had dropped to 20/200 in both eyes. At slit lamp examination, a mild inflammation of the anterior segment was present, the IOL was well positioned in the capsular bag, and dense vitreitis veiled the fundus. Intraocular pressure was 18 mmHg in both eyes.

She returned to her ophthalmologist and underwent repeated cycles of topical, subconjunctival and subtenon injections of steroids (Betamethasone phosphate), as well as mydriatic and cyclopegic eye drops. To rule out other potential causes of uveitis, a comprehensive workup was performed, but all tests were unremarkable.

After three months of steroid therapy, the lack of improvement led the ophthalmologist to prescribe systemic steroid therapy (Prednisone 1 gr/Kg/day) but the patient gained no benefit even from this therapy. At this time, her visual acuity had dropped to light perception in both eyes, and the IOP had reached 35 mmHg in the right eye and 50 mmHg in the left eye. Antiglaucomatous therapy was then prescribed (topical Beta blockers and topical and systemic carbonic anhydrase inhibitors) and, on the suspicion of a steroid-induced glaucoma, systemic steroid therapy was stopped and the patient was referred to an immunologist. She was then prescribed Cyclosporine A at the dosage of 3 mg/Kg/die; but despite this therapy her sight did not improve, the IOP did not decrease, and she developed hypertension and renal failure.

This unfortunate doctor then went to a new ophthalmologist, who performed a pars plana vitrectomy and a trabeculectomy in her left eye; but, despite a clear vitreous chamber, her left eye visual acuity did not improve because a dense inflammatory reaction developed in the anterior chamber (Figure 1(b)), while the IOP decreased for a few days then returned to 50 mmHg despite therapy. 

She consulted a new immunologist, who changed the immunosuppressive therapy, prescribing prednisone 1 mg/Kg/day and MTX 20 mg/week. It was at this time (December 2010) that the patient came to our attention.

Her visual acuity was light perception in both eyes, the IOP was 35 mmHg RE and 50 mmHg LE, despite topical and systemic antiglaucomatous therapy. In the RE a dense vitreitis hindered fundus examination (Figure 1(a)), and a pale optic nerve was scarcely visible at the fundus examination of the LE. An intense granulomatous inflammation was present in the anterior chamber of the left eye (Figure 1(b)). At this time, the patient began to show personality changes: she became oblivious to the environment and did not seem to attribute any importance to the severity of her visual defect. Despite the new systemic immunosuppressive therapy, no improvement was obtained. 

Suddenly, one day, the patient fainted. She was immediately taken to the ER, where she underwent MRI (Figure 1(e)). MRI of the brain showed a hypointense multifocal lesion occupying much of the right frontal and temporal lobe, the right occipital lobe, and the left parietal lobe. In T1-weighted MRI images, the lesion was hypointense while at T2-weighted images, it was hyperintense with contrast enhancement. The lesions showed cystic foci after gadolinium injection, with a ring-like enhancement representing central necrosis. Intense peritumoral edema was present. 

A diagnosis of PCNSL was suspected and a stereotactic biopsy was performed.

## 3. Pathological Findings

**Figure 2:**
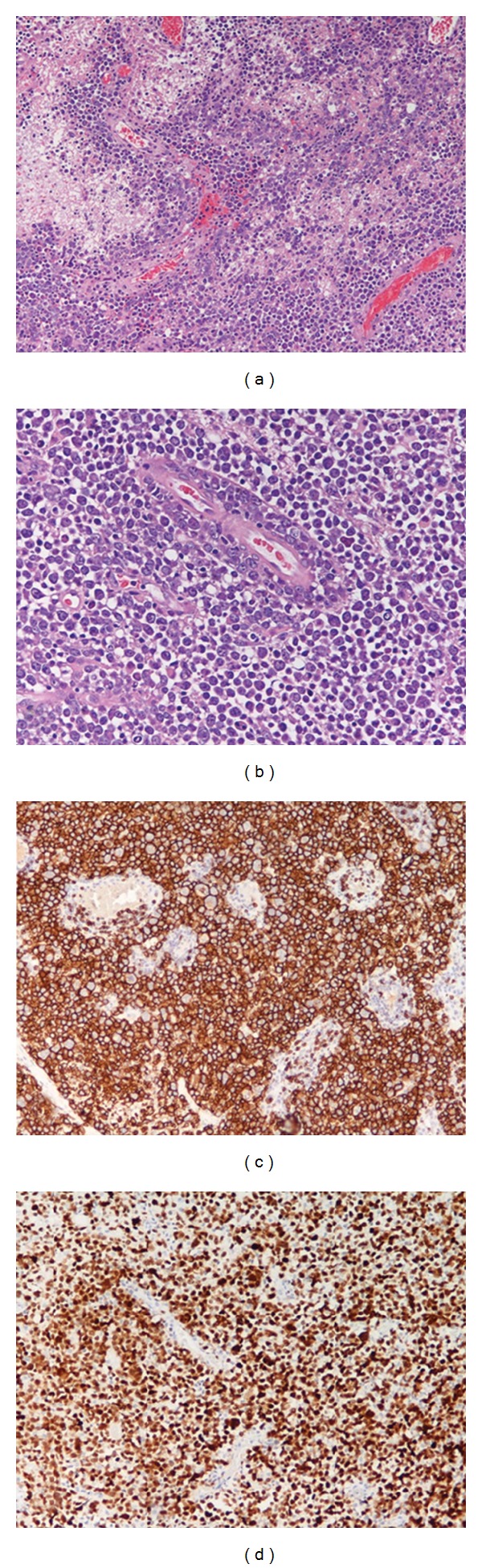
(a) Areas of necrosis associated to perivascular islands of viable lymphoma cells (hematoxylin and eosin; original magnification ×200), (b) Characteristic perivascular accumulation and spread of blastic lymphoma cells (hematoxylin and eosin; original magnification ×100). (c) PCNSL cells display consistent CD20 immunoreactivity (original magnification ×100). (d) PCNSL cells display consistent MUM-1 immunoreactivity (original magnification ×100).

Histological examination of the specimen showed neoplastic blastic cells arranged in dense cellular aggregates with prevalent diffuse growth pattern and with large pleomorphic nuclei and distinct nucleoli (Figure 2(a)). Several areas of coagulative necrosis were observed in the middle of perivascular islands of viable tumor cells. Typical angiocentric infiltration pattern was noted (Figure 2(b)). The proliferation index was very prominent. The cells demonstrated consistent immunoreactivity for CD20 and MUM-1 (Figure 2(c)). No immunoreactivity for CD3, CD138, and Glial fibrillary acidic protein (GFAP) was detected in the cells (Figure 2(d)). A diagnosis of primary CNS diffuse large B-cell lymphoma was made. 

The patient then underwent chemo- and radio-therapy: First-line therapy was based on systemic chemotherapy with high-dose methotrexate (5 g/m^2^) in combination with Ara-C (cytarabine) (2 g/m^2^) twice daily for two cycles, followed by whole brain radiation therapy (WBRT) with a dosage of 24–36 Gy. 

The intraocular inflammation resolved. Her best corrected visual acuity is now 60/200 in the RE and 20/200 in the LE. At fundus examination, a bilateral optic glaucomatous subatrophy is present, and the vitreous floaters have completely disappeared. The IOP decreased in both eyes to 16 mmHg without any therapy, probably because it was secondary to blockage of the trabecular meshwork by the lymphoma cells in the anterior chamber.

## 4. Discussion

The term “Masquerade syndrome” is used for malignant diseases manifesting as peri- or intraocular inflammations of unknown origin (iritis, vitritis, uveitis) [1].

One of the following diagnoses must be suspected in all cases of ocular inflammation unresponsive to classical therapy: primary central nervous system lymphoma (PCNSL), primary intraocular lymphoma (PIOL), choroid melanoma, retinoblastoma, intraocular metastases, para-neoplastic retinopathy [2]. Patients complain of increasingly blurred vision. This is either due to inflammation of the anterior chamber (iritis), or, more commonly, to an infiltration of the vitreous body (vitritis, uveitis), the retina, and the choroid. Patients may also complain of so-called “floaters.” A reduction in visual acuity and even amaurosis can occur. These changes may be limited to just one eye. Primary central nervous system lymphoma, on the other hand, causes bilateral involvement in 80% of cases [3, 4].

A prompt diagnosis is very important. A potential delay in reaching a diagnosis arises as a masquerade syndrome frequently responds to corticosteroids which mask the symptoms.

Making the right diagnosis has vital consequences for patients as most cases are malignant. PCNSL, in particular, should be considered in the differential diagnosis in middle-aged patients as it is the commonest cause of a masquerade syndrome. In order to differentiate between other causes of masquerade syndrome, in addition to a careful history and clinical examination, the following investigations are essential: ultrasound [5], fluorescent angiography, neuroimaging [6], CSF cytology and finally a vitrectomy [7], or chorioretinal biopsy with immunohistological examination [8].

Our patient was particularly unlucky: despite the signs that might suggest a masquerade syndrome, no physician suspected the underlying disease, nor were immunohistological and immunohistochemical examinations of the specimen performed at the time of the vitrectomy.

Furthermore, the prolonged steroid therapy certainly contributed to hide the underlying disease, and the immunosuppressive therapy probably facilitated the multicentric manifestation of the lymphoma, as in immunocompromised patients.

All ophthalmologists and clinicians must be aware of masquerade syndromes, in order to avoid a delay in diagnosis, as occurred in this case of our unlucky colleague.
